# Synergy between conventional antibiotics and anti-biofilm peptides in a murine, sub-cutaneous abscess model caused by recalcitrant ESKAPE pathogens

**DOI:** 10.1371/journal.ppat.1007084

**Published:** 2018-06-21

**Authors:** Daniel Pletzer, Sarah C. Mansour, Robert E. W. Hancock

**Affiliations:** Centre for Microbial Diseases and Immunity Research, Department of Microbiology and Immunology, University of British Columbia, Vancouver, Canada; Harbor-UCLA Medical Center, UNITED STATES

## Abstract

With the antibiotic development pipeline running dry, many fear that we might soon run out of treatment options. High-density infections are particularly difficult to treat due to their adaptive multidrug-resistance and currently there are no therapies that adequately address this important issue. Here, a large-scale *in vivo* study was performed to enhance the activity of antibiotics to treat high-density infections caused by multidrug-resistant Gram-positive and Gram-negative bacteria. It was shown that synthetic peptides can be used in conjunction with the antibiotics ciprofloxacin, meropenem, erythromycin, gentamicin, and vancomycin to improve the treatment outcome of murine cutaneous abscesses caused by clinical hard-to-treat pathogens including all ESKAPE (*E**nterococcus faecium*, *S**taphylococcus aureus*, *K**lebsiella pneumoniae*, *A**cinetobacter baumannii*, *P**seudomonas aeruginosa*, *E**nterobacter cloacae*) pathogens and *Escherichia coli*. Promisingly, combination treatment often showed synergistic effects that significantly reduced abscess sizes and/or improved clearance of bacterial isolates from the infection site, regardless of the antibiotic mode of action. *In vitro* data suggest that the mechanisms of peptide action *in vivo* include enhancement of antibiotic penetration and potential disruption of the stringent stress response.

## Author summary

There has been enormous publicity about the inexorable rise of resistance and the dearth of new therapies. However less attention has been placed on adaptively multidrug-resistant high density bacterial infections for which antibiotics are highly used but no effective therapies currently exist. Here we have provided new hope for this previously intractable class of infections as typified by abscess infections that are responsible for 3.2 million annual emergency room visits in the US alone. We show how to enhance the activity of antibiotics to treat multidrug-resistant Gram-positive and Gram-negative bacteria, using peptides that target the bacterial stress response, persister-based resistance and the outer membrane permeability barrier. In particular we have employed a new bacterial subcutaneous abscess mouse model to demonstrate that: (a) 7 of the society’s most recalcitrant pathogens formed cutaneous abscesses and even when antibiotics were directly delivered into abscess tissues, they showed poor efficacy; (b) By combining antibiotics with the local administration of anti-biofilm peptides that target cellular (stringent) stress responses, we could pharmacologically treat the infection and reduce the severity of cutaneous abscesses; (c) This synergy was due to increased outer membrane permeability as well as the disruption of the conserved stringent stress response that controls virulence and antibiotic resistance, particularly due to so-called persisters. These peptides have therefore the potential to broaden our limited antibiotic arsenal for a group of extremely difficult to treat infections.

## Introduction

ESKAPE pathogens (*E*. *faecium*, *S*. *aureus*, *K*. *pneumoniae*, *A*. *baumannii*, *P*. *aeruginosa*, *E*. *cloacae*) are recognized to be responsible for the majority of difficult-to-treat community-acquired, healthcare-associated, and nosocomial infections [1]. Multidrug-resistant bacteria represent major therapeutic challenges and pose a great threat to human health [2]. The increasing resistance to available antibiotics dampens treatment possibilities and there is a serious lack of adequate treatment options. Less discussed but of even greater concern are infections associated with high bacterial densities (>10^7^ CFU/ml bacteria) especially biofilm and/or abscess infections. High bacterial densities lead to elevated MICs to multiple antibiotics [3] and are extremely difficult to treat with antibiotics [4]. In this context, skin and soft tissue infections (SSTIs) are an emerging problem, a significant burden in health care facilities, and responsible for increased antibiotic administration [5]. SSTIs such as abscesses form fluid, pus-filled pockets infiltrated by bacteria and immune cells [6], and are often highly resistant to antibiotic treatment. Indeed, abscesses are the most common indication for frequent (6–12 h), high-dose (up to 1 g/kg) and long term (>5 d) [7] intravenous (IV) broad-spectrum antibiotic administration [5]. SSTIs have been traditionally thought to be largely caused by *S*. *aureus* and *Streptococcus pyogenes* but recent findings show that other microbes are very prevalent [8, 9]. Indeed, the SENTRY antimicrobial surveillance program (North America) [10] reported that the major pathogens isolated from SSTIs now include 10.8% *P*. *aeruginosa*, 8.2% *Enterococcus* sp., 7.0% *E*. *coli*, 5.8% *Enterobacter* sp., and 5.1% *Klebsiella* sp., as well as 45.9% *S*. *aureus*. Moreover, recently *A*. *baumannii* is increasingly recognized as an emerging cause of nosocomial infections and important cause of severe, life-threatening soft tissue infections [11]. High bacterial numbers of greater than 10^8^ CFU/ml isolated bacteria are present in soft-tissue and peritoneal infections [12], highlighting the importance of investigating high-bacterial density infections. However, standard *in vitro* susceptibility tests employ modest bacterial concentrations of 2–5 x 10^5^ per ml which critically underestimates the strong impact on antibiotic susceptibility of the high concentrations of bacteria in such infections [12]. Thus, it remains a major challenge to translate *in vitro* findings into *in vivo* efficacy and compounds that show excellent *in vitro* activity (e.g., low MIC in defined medium), often work poorly when tested under *in vivo* conditions.

## Results and discussion

**Fig 1:**
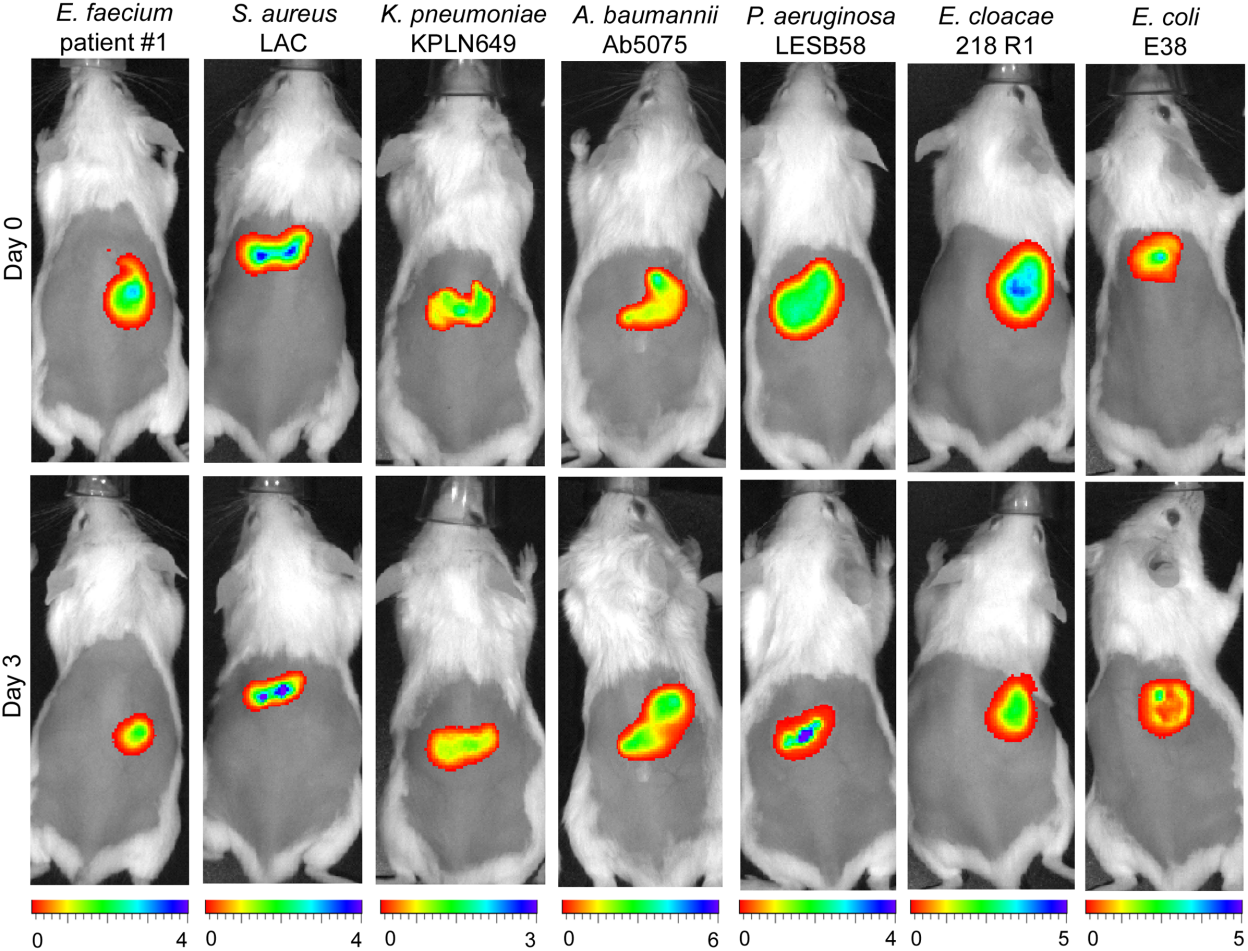
*In vivo* tracking of bioluminescently labeled (live) bacterial infections. CD-1 female mice were injected with individual bacterial strains carrying plasmids constitutively expressing *lux* reporter genes. Bacterial strains were injected subcutaneously at a dose of 1 × 10^9^ CFU *E*. *faecium*, 5 × 10^7^ CFU *S*. *aureus*, 1 × 10^9^ CFU *K*. *pneumoniae*, 1 × 10^9^ CFU *A*. *baumannii*, 5 × 10^7^ CFU *P*. *aeruginosa*, 2.5 × 10^8^ CFU *E*. *cloacae*, and 1 × 10^8^ CFU *E*. *coli*. The infection was monitored 1 h post infection and then every 24 h until day 3. Representative images for day 0 and day 3 are shown. Mice were imaged using a Perkin Elmer *in vivo* imaging system (IVIS) and the experiment was repeated twice with three mice/group. The scale at the bottom indicates radiance x 10^6^.

To investigate abscess infections caused by the ESKAPE pathogens and *E*. *coli*, we extrapolated from our previously-developed cutaneous mouse infection model [4], prioritizing the study of resistant, recalcitrant host-adapted pathogens rather than commonly used laboratory strains. We identified clinical isolates that were able to cause chronic skin abscesses on the backs of CD-1 female mice after injection of a high bacterial dose (≥ 10^7^ bacteria); each of these strains persisted throughout the course of a three day experiment and did not cause mortality in mice. MIC assays revealed that these strains had generally low antibiotic susceptibility and were resistant to antibiotics from at least three different classes (Table 1, S1 Table). Plasmid-encoded bioluminescently-tagged isolates were created to enable visualization and monitoring of the progress of disease using non-invasive techniques, and to provide evidence that the skin infection contained metabolically active bacteria; this enabled us to follow the infection for all strains (Fig 1).

**Table 1:** *In vitro* MICs of antibiotics and peptides against clinical isolates.

Strain	MIC (μg/ml)															
	AZA^a^	AZM	CAM	CAZ	CIP	COL	ERY	GEN	MERO	RIF	TOB	VAN	1002	HHC-10	1018	DJK-5
*A*. *baumannii* Ab5075	>500	15.6	125	>500	62.5	0.2	**15.6**^b^	>500	**15.6**	1.6	250	62.5	23.1	23.1	23.1	**11.6**
*E*. *coli* E38	0.1	7.8	<3.9	<3.9	**0.02**	0.3	62.5	12.5	0.1	12.5	2.5	250	4.6	4.6	**9.3**	**2.3**
*E*. *cloacae* 218R1	62.5	7.8	7.8	62.5	**0.04**	0.3	250	3.1	0.6	12.5	0.3	250	23.1	**23.1**	**4.6**	9.3
*E*. *faecium* #1–1	>500	>500	25	62.5	250	>500	>500	**15.6**	>250	12.5	500	500	**4.6**	7.4	**4.6**	2.3
*K*. *pneumoniae* KPLN649	>500	3.9	15.6	62.5	**6.3**	0.6	500	>500	**0.1**	>500	125	>500	37	>46.3	**18.5**	**23.1**
*P*. *aeruginosa* LESB58	250	62.5	7.8	15.6	**3.1**	6.3	250	250	3.1	25	31.3	250	46.3	18.5	**46.3**	**46.3**
*S*. *aureus* LAC	>500	250	7.8	62.5	5	125	31.3	3.1	1.6	<0.4	<0.4	**1.6**	<0.4	46.3	148	**37**

^a^ AZA, Aztreonam; AZM, Azithromycin; CAM, Chloramphenicol; CAZ, Ceftazidime; CIP, Ciprofloxacin; COL, Colistin; ERY, Erythromycin; GEN, Gentamicin; MERO, Meropenem; RIF, Rifampicin; TOB, Tobramycin; VAN, Vancomycin

^b^ bold numbers indicate the antibiotics/peptides used in this study

**Fig 2:**
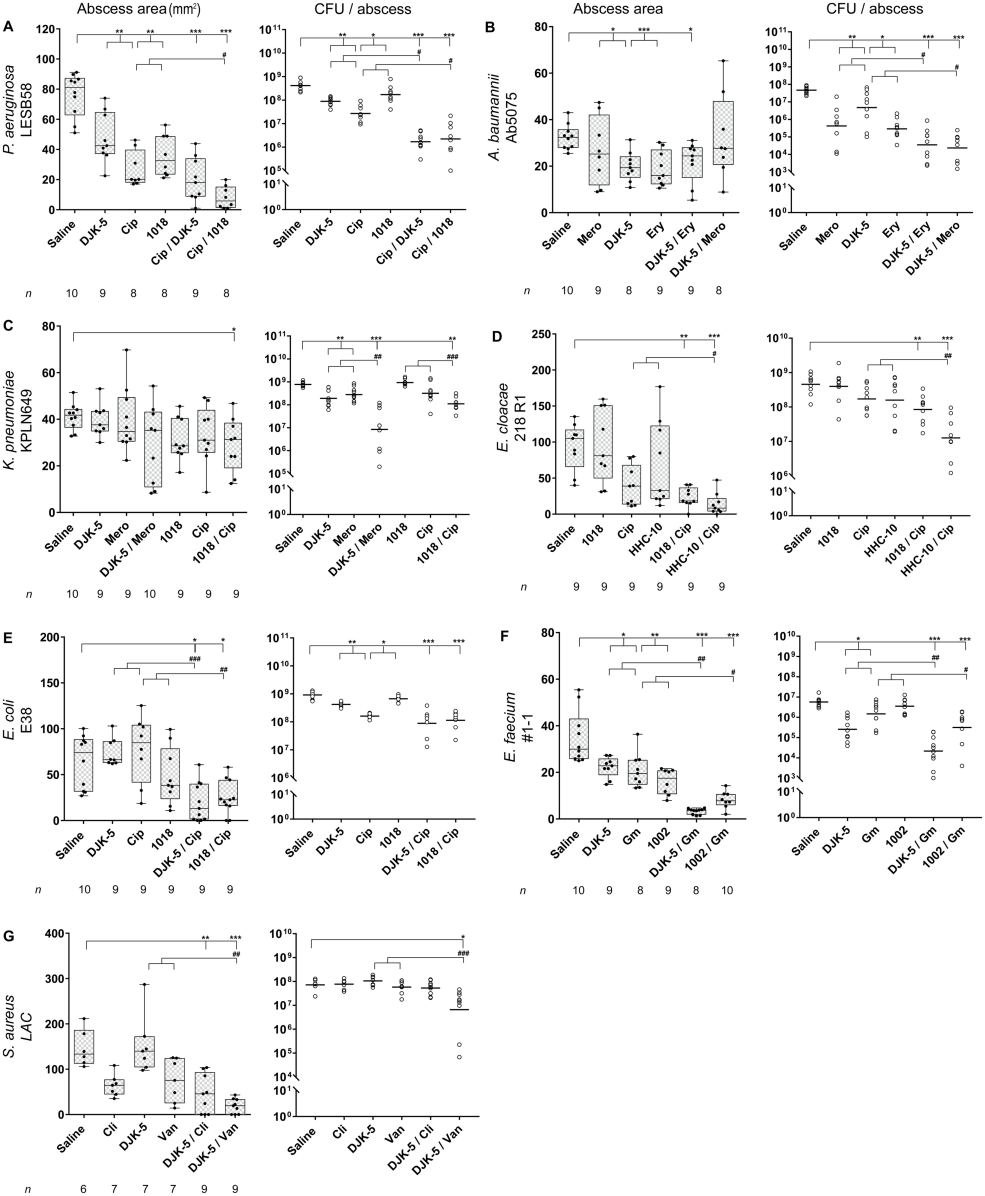
Antibiotic and synthetic peptide mono- and combinatorial therapy in a murine cutaneous abscess model using female CD-1 mice and clinical drug-resistant bacterial isolates. Bacterial strains were injected subcutaneously and treated one hour post infection with either saline (control), synthetic peptides, antibiotics, or antibiotic-peptide combinations. Synthetic peptide concentrations for all conditions were as follows: 1002, 10 mg/kg (3 mg/kg for *E*. *faecium*); 1018, 10 mg/kg; HHC-10 10 mg/kg, and DJK-5, 3 mg/kg (0.25 mg/kg for *S*. *aureus*). Infected and inflamed tissue was measured three days post infection and pus-containing abscess lumps excised to determine CFU. Abscess sizes are in box and whiskers plots (left panel) and counted CFU/abscess data expressed with geometric mean (right panel). **A**. *P*. *aeruginosa* LESB58, ciprofloxacin 0.4 mg/kg. **B**. *A*. *baumannii* Ab5075, erythromycin 6 mg/kg, meropenem 6 mg/kg. **C**. *K*. *pneumoniae* KPLN649, meropenem 10 mg/kg, ciprofloxacin 30 mg/kg. **D**. *E*. *cloacae* 218 R1, ciprofloxacin 0.006 mg/kg. **E**. *E*. *coli* E38, ciprofloxacin 4 mg/kg, F. *E*. *faecium* #1–1, gentamicin 16 mg/kg. G. *S*. *aureus* LAC, clindamycin 0.01 mg/kg; vancomycin 0.15 mg/kg. **(A-G)** *n* = 368 biologically independent animals. All experiments were done at least three times with 2–4 mice/group. Statistical analysis was performed using One-way ANOVA, Kruskal-Wallis test with Dunn’s correction (two-sided). The asterisk indicates significant differences to the wild-type (*, *p* < 0.05; **, *p* < 0.01; ***, *p* < 0.001). The hash indicates significant differences of the combination therapy over the sum of the effects of each agent alone (#, *p* < 0.05; ##, *p* < 0.01; ###, *p* < 0.001).

To optimize the treatment strategy, antibiotics were chosen based on their moderate *in vitro* MIC values (0.02 to 15.6 μg/ml) and empirically tested *in vivo* to determine an appropriate concentration that reduces abscess sizes and/or CFU just enough to observe synergy of the peptides and antibiotics. Since the drug concentrations after IV injection might be affected by various factors including blood perfusion, penetration into tissues and/or binding to plasma proteins or dermal components [13], we directly injected antibiotics into the infected tissue. This allowed us to overcome the distinct pharmacokinetics of antibiotics used in humans as opposed to mice when applied intravenously, as well as the amounts delivered and time for penetration (S1 Table) to the target site [13, 14]. For each antibiotic directly injected into the infected abscess tissue, an amount that would provide a total body concentration greater than the effective antibiotic dose was chosen. Meropenem was used to treat *A*. *baumannii* and *K*. *pneumoniae* infections at concentrations of 6 and 10 mg/kg, respectively. The MIC for meropenem against *K*. *pneumoniae* was very low (0.1 μg/ml), while the MIC against *A*. *baumannii* was quite high (15.6 μg/ml). Based on EUCAST clinical breakpoint information, *K*. *pneumoniae* KPLN649 is resistant to meropenem while *A*. *baumannii* Ab5075 is sensitive (S1 Table). However intriguingly, treatment of an *A*. *baumannii* infection reduced bacterial cell numbers by 111-fold, while there was only a 2.7-fold clearance of *K*. *pneumoniae*. Indeed, *K*. *pneumoniae* showed the highest recalcitrance towards all tested antibiotic treatments in this skin infection model and high concentrations of azithromycin (500 mg/kg) or colistin (3 mg/kg) had no anti-infective activity. Similarly, although the MICs of ciprofloxacin against *P*. *aeruginosa* (3.1 μg/ml) and *K*. *pneumoniae* (6.3 μg/ml) were quite similar, and both strains resistant to ciprofloxacin based on EUCAST (S1 Table), as little as 0.4 mg/kg ciprofloxacin were required to reduce the *P*. *aeruginosa* load by 15-fold, while a 75-fold greater dosage of 30 mg/kg reduced the *K*. *pneumoniae* bacterial burden by only 2-fold. Similarly, the *E*. *coli* and *E*. *cloacae* strains were sensitive towards ciprofloxacin (S1 Table), with MICs 0.02 and 0.04 μg/ml, respectively; however, in the mouse abscess model 4 mg/kg was required to reduce *E*. *coli* cells by 5.8-fold while only 0.006 mg/kg was required to reduce *E*. *cloacae* by 2.8-fold (Fig 2A–2E) (S2 Table).

These important observations highlight that antibiotic mono-therapies as well as high antibiotic dosages are often ineffective when bacteria form high density infections. Thus *in vitro* MICs are useful indicators of potential, but are not always predictive of *in vivo* efficacy especially when adaptive resistance occurs. The situation was even more difficult for strains that were resistant towards several classes of antibiotics, e.g. an *E*. *faecium* patient isolate that showed extensive drug resistance towards all tested antibiotics (Table 1). In this case the use of gentamicin for *in vivo* treatment at a high dosage (16 mg/kg) led to a reduction of the bacterial burden by about 4-fold (Fig 2F, S2 Table). Conversely, a methicillin resistant *S*. *aureus* infection was somewhat treatable, although sensitive to the used antibiotics based on EUCAST (S1 Table), with clindamycin (0.01 mg/kg) and vancomycin (0.15 mg/kg), both of which visually reduced abscess sizes, but had no impact on bacterial clearance (Fig 2G, S2 Table).

Host defense peptides (HDPs) are small cationic amino acids groups produced by the body as a defense mechanism and are key components of immunity [15], while short synthetic derivatives show promise as broad spectrum anti-infectives that protect in various animal models [16]. A discrete subset of these peptides is effective against a broad spectrum of bacterial biofilms [17]. The mechanism of action of such peptides, e.g. 1018 and DJK-5, has been linked to the disruption of the stringent stress response [17, 18]. The bacterial stringent response is a highly conserved (present in Gram-positive and Gram-negative bacteria) response to various stresses that impacts virulence and antibiotic susceptibility. Their unique ability to disrupt this stress response enables such peptides to show activity against stringent response controlled abscess infections, where biofilm phenotypes have also been suggested to play a crucial role [17, 19, 20]. In this context, although our peptides showed high MICs *in vitro* (Table 1), we previously showed that they can reduce abscess sizes as well as have modest effects in reducing bacterial numbers when *S*. *aureus* or *P*. *aeruginosa* infections were treated [18, 20].

Here we hypothesized that they would convert bacteria into a more relaxed (unstressed) state that would render them more susceptible to antibiotic treatment. In this regard, peptide DJK-5 at just 3 mg/kg reduced bacterial loads of *P*. *aeruginosa* (4.6-fold), *E*. *faecium* (22-fold), *K*. *pneumoniae* (4.0-fold), *A*. *baumannii* (9.9-fold), and *E*. *coli* (2.2-fold) infections (Fig 2A–2C and 2E, S2 Table). Other peptides, namely 1002, 1018, and HHC-10 at concentrations of 10 mg/kg had no significant impact on the bacterial burden but showed promise in visually reducing abscess sizes for *P*. *aeruginosa*, *E*. *faecium*, *K*. *pneumoniae*, *E*. *coli*, and *E*. *cloacae* infections (Fig 2A, 2C–2F).

Antibiotic combination therapy is frequently used as a possible method of outmaneuvering recalcitrant bacterial pathogens [21] but its application remains controversial and debated, in part due to the increased risk of toxicity, organ damage, and the selection and emergence of resistant strains [22]. Unfortunately, although *in vitro* assessments of synergy employing checkerboard titration have been used to justify combination therapy, these are rarely followed up with animal model infection studies. Testing synthetic peptides in combination with antibiotics *in vivo* showed the ability to enhance the treatment outcome of multidrug resistant bacterial infections. *In vivo* synergy was defined as demonstrating an effect that was significantly more pronounced in the combination than the sum of the effects of each agent alone using saline-treated animals as a negative control [23, 24]. By this definition, combination therapies applied with DJK-5 and antibiotics significantly worked against *P*. *aeruginosa* (reduction of bacterial load by 245-fold in combination with ciprofloxacin), *E*. *faecium* (265-fold with gentamicin), *K*. *pneumoniae* (91-fold with meropenem), *A*. *baumannii* (1325-fold with erythromycin and 2006-fold with meropenem), and *S*. *aureus* (11-fold with vancomycin) when comparing bacterial burdens to the saline control. Indeed DJK-5 worked synergistically and showed an average overall improvement of 14-fold compared to the sum of the individual single treatments. The highest synergistic activity occurred when DJK-5 was combined with meropenem or erythromycin against *A*. *baumannii*, reducing the bacterial load by 65-fold and 33-fold, respectively, in combination treatment compared to the sum of individual treatments (Fig 2B; S2 Table). For *E*. *coli*, DJK-5 in combination with ciprofloxacin showed a 10.3-fold reduction in bacterial numbers over the saline control, which was still a 2.9-fold improvement over the summed monotherapies (Fig 2E).

Analogous data was also obtained with other peptides; 1018 in combination with ciprofloxacin showed about 186-fold, 6.9-fold, 5.4-fold, and 8-fold reduction in bacterial burdens against *P*. *aeruginosa*, *K*. *pneumoniae*, *E*. *cloacae*, and *E*. *coli* over the saline control (Fig 2A and 2C–2E) and additionally was synergistic in reducing *P*. *aeruginosa* and *K*. *pneumoniae* numbers from the abscess tissue by 30- and 6.4-fold, respectively compared to the summed monotherapies (Fig 2A and 2C). The combination of HHC-10 with ciprofloxacin against *E*. *cloacae* reduced the bacterial load by about 36-fold compared to the saline control and showed synergistic effects over the summed monotherapies (13-fold enhanced activity) (Fig 2D). Gentamicin in combination with peptide 1002 against *E*. *faecium* showed an 18-fold reduction in comparison to the control, and a synergistic effect over the sums of the single administrations (7.1-fold increased reduction in bacterial burden) (Fig 2F). Thus, although peptides 1002, 1018, and HHC-10 appeared to be less active in combination treatments, possibly due to their being composed of L-amino acids that makes them susceptible to host proteases, they were also shown to work synergistically under *in vivo* conditions.

Although genetically-determined antibiotic resistance has been well publicized as a major issue in human health, the current inability to deal with phenotypic multi-drug resistance (e.g. engendered due to growth conditions, especially biofilms), and in particular high-density infections, has not been well addressed. Here, we examined peptides as a possible adjuvant to antibiotic therapy for treating high-density, recalcitrant bacterial abscess infections caused by the most intractable bacterial species. Our strategy to combine conventional antibiotics with synthetic peptides offers a novel therapeutic approach to effectively treat high density infections in that a range of combination therapies were able to reduce the bacterial burden of problematic clinical isolates in our subcutaneous infection model. At least part of the effect of peptides on antibiotic action was likely due to the ability of peptides to elicit degradation of the stringent stress response intracellular signaling molecule ppGpp [17, 25], which has been tied to resistance induction [26, 27], and the development of energy-starved persisters [28, 29]. To further elucidate how the peptides acted, we performed checkerboard titration experiments to determine interactions between the antibiofilm peptides 1018 and DJK-5 and the antibiotic ciprofloxacin against *P*. *aeruginosa* LESB58. Since antibiofilm peptides exert their activities under stringent stress conditions [17, 25], such as encountered in biofilms or abscesses, the stringent response (ppGpp) inducing agent serine hydroxamate (SHX) as well as a ppGpp-overproducing strain (LESB58 containing the overexpressed cloned *relA* gene) were used (S3 Table). As expected, under planktonic growth conditions there was no effect of the combined treatment. However, for the two ppGpp-overexpressing situations, combining the two agents lowered the effective concentrations of each agent to below the MIC, but this was prevented in a stringent response Δ*relA*Δ*spoT* double mutant. The insertion of the cloned *relA* gene into the double mutant enabled effective ciprofloxacin-peptide combinations. In this context, the mutant lacking the stringent response genes, was 2-fold more susceptible towards ciprofloxacin while the mutant complemented with the ppGpp synthetase, RelA, had a 4-fold higher MIC to ciprofloxacin (S3 Table). Stress related responses could provide another mechanism of synergy in addition to peptide-mediated breach of the Gram-negative permeability barrier. These *in vitro* findings provide a plausible mechanism that may also be occurring *in vivo*.

Most available antibiotics target intracellular processes and therefore must penetrate the bacterial cell envelope, which is particularly challenging in Gram-negative bacteria due to their formidable outer membrane. To further investigate the molecular basis of the synergistic and additive effects of the combinatorial treatment, we performed outer membrane permeability assays, observing the ability of peptides to enhance the uptake of the normally impermeable hydrophobic fluorophore N-phenyl-1-naphtylamine (NPN). Colistin, a cationic lipopeptide antibiotic that is known to permeabilize the bacterial outer membrane [30], served as a positive control, while meropenem, gentamicin, or vancomycin were used as negative controls. Except for 1002, each peptide was able to interact with and permeabilize the outer membrane of Gram-negative bacteria at their corresponding MICs (Table 2) and/or 10xMICs (S4 Table). The effect of the peptides on Gram-positive bacteria was almost undetectable since NPN has almost unimpeded access to Gram-positive bacteria.

**Table 2:** Outer membrane permeabilization by peptides cf. antibiotics at their corresponding MICs. The uptake of the fluorophore NPN in the presence of different antibiotics and synthetic peptides was determined by assessing increased fluorescence at an excitation wavelength of 350 nm and an emission wavelength of 420 nm due to partition of the normally impermeable hydrophobic NPN into bacterial membranes. Relative fluorescence values of at least three biological replicates were determined by subtracting the fluorescence value without test substance.

Strain	Relative NPN Fluorescence (Mean ± Standard Error)					
	Antibiotic^a^	1018	HHC-10	DJK-5	1002	Colistin
*A*. *baumannii* Ab5075	0 ± 0	145 ± 9^d^	120 ± 6^d^	48 ± 14	85 ± 29	56 ± 49
*E*. *coli* E38	1.4 ± 1.4	72 ± 6^d^	69 ± 12^d^	21 ± 3.5	6.0 ± 0.9	12 ± 1.2
*E*. *cloacae* 218R1	4.5 ± 3.4	26 ± 11	102 ± 12^d^	12 ± 7.2	9.3 ± 4.7	0.3 ± 0.3
*K*. *pneumoniae* KPLN649	0 ± 0	33 ± 12^d^	116 ± 36^d^	13 ± 0.7	25 ± 3.5	48 ± 36
*P*. *aeruginosa* LESB58	4.5 ± 4.5	275 ± 110^d^	72 ± 8	140 ± 29^d^	113 ± 49	210 ± 57^d^
*E*. *faecium* #1–1	2.6 ± 2.3^b^	1.9 ± 1.2	0 ± 0	0.6 ± 0.6	0 ± 0	0 ± 0
*S*. *aureus* LAC	2.9 ± 2.9^c^	7.1 ± 5.7	0 ± 0	1.8 ± 1.8	8.2 ± 2.5	9.7 ± 5.5

^a^ Meropenem was used unless indicated otherwise

^b^ Gentamicin

^c^ Vancomycin

^d^ Significant difference to the antibiotic (*p* < 0.05; One-Way ANOVA, Kruskal-Wallis test).

The peptides used here worked with a variety of antibiotics. However, future work should explore further possible combinations to find those most optimal. Other possible mechanisms contributing to synergy should be investigated including modulating the host innate immune/inflammatory responses (increasing protective responses while dampening inflammation) and yet-to-be-identified downstream processes associated with the blockage of the stringent response.

The insights from our study could help physicians to understand bacterial infections in skin and soft tissues, and aid in management and development of improved treatment strategies. Ultimately, we have provided evidence that our peptides, especially DJK-5, showed superior effects when paired with antibiotics.

## Materials and methods

### Bacterial strains and growth conditions

Bacterial strains used in this study were *E*. *faecium* #1–1 (BEI resources, NR-31903), *K*. *pneumonia* KPLN649 [31], *A*. *baumannii* Ab5075 [32], *P*. *aeruginosa* LESB58 [33], *E*. *cloacae* 218R1 [34], *E*. *coli* E38 (Serotype O78:H-) (BEI resources, NR-17717), and *S*. *aureus* LAC USA300 [35] (S5 Table). All organisms were cultured at 37°C in double Yeast Tryptone (dYT). Liquid cultures were grown at 37°C with shaking at 250 rpm. Cultures harboring individual plasmids were supplemented with 15 μg/ml gentamicin (Gm), 100 μg/ml ampicillin (Ap), 10 μg/ml tetracycline (Tc), 25 μg/ml chloramphenicol (Cm), 100 μg/ml spectinomycin (Sp) for *E*. *coli*, 500 μg/ml Gm for *P*. *aeruginosa*, 100 μg/ml Cm for *K*. *pneumoniae*, 25 μg/ml Tc for *A*. *baumannii*, 25 μg/ml Gm for *E*. *cloacae*, and 400 μg/ml Sp for *E*. *faecium*. Bacterial growth was monitored using a spectrophotometer at an optical density of 600 nm (OD_600_).

### Construction and transformation of bioluminescent plasmids

The 5.8-kb *luxCDABE* operon was PCR amplified from pUCP.lux using the primer lux-fd (CCGCAAATGGATGGCAAATA) and transcriptional terminator t0-containing primer lux-rv-t0 (TGGACTCACAAAGAAAAAACGCCCGGTGTGCAAGACCGAGCGTTCTGAACAATCAACTATCAAACGCTTCGG). The resulting PCR fragment was cloned into the pCR-BluntII-TOPO vector and successful transformants isolated based on their expression of luminescence. A broad-host-range cloning vector, pBBR1MCS [36], with various antibiotic resistance markers to constitutively express luminescence genes from the *lac* promoter was utilized by transferring the complete *luxCDABE-t0* fragment into pBBR1MCS-1, pBBR1MCS-3, or pBBR1MCS-5, via *Kpn*I / *Pst*I restriction sites (S6 Table). Successful transformants were selected based on luminescence expression and further verified by restriction digestion.

*P*. *aeruginosa* LESB58 and *A*. *baumannii* Ab5075 were made electrocompetent with 300 mM sucrose. *E*. *cloacae* 218R1, *K*. *pneumonia* KPLN649, *E*. *faecium* #1–1, and *E*. *coli* E38 were made electrocompetent with ice-cold water. Briefly, individual strains except *E*. *faecium* were scraped from an overnight grown agar plate and washed with either sucrose or water. *E*. *faecium* was scraped from a plate and grown overnight in dYT supplemented with 3% glycine. Electroporation conditions were 2.5 kV, 25 μF, 200 Ω. Plasmid pUCP.lux was transformed into *P*. *aeruginosa*, pBBR1.lux into *K*. *pneumoniae*, pBBR3.lux into *A*. *baumannii* and *E*. *coli*, pBBR5.lux into *E*. *cloacae*, and plasmid pSL101-P16S, which has a broad-host range replicon for Gram-positive bacteria, into *E*. *faecium*. Successful transformants were checked for luminescence expression and plasmid stability further verified by re-streaking single colonies on agar plates without antibiotic selection for 4 days, which did not lead to the loss of luminescence signals.

### Construction of the *relA* overexpression plasmid

A 2447-bp fragment containing the *relA* gene including a 100-bp upstream promoter region was PCR amplified from *P*. *aeruginosa* LESB58 genomic DNA using the primers relA_oe_fwd-Spe (CATACTAGTGGGTATCTCGGGTCTTCAG) and relA_oe_rev-Apa (TCAGGGCCCGCTAGGATGCCTGCGTAATC). The resulting PCR fragment was cloned into pBBR1MCS-5 [36] via *Spe*I and *Apa*I restriction sites and sent for sequencing before transformation into *P*. *aeruginosa* LESB58.

### Peptide synthesis, antibiotics, and *in vivo* application

Peptides HHC-10 (KRWWKWIRW-NH_2_) [37], 1002 (VQRWLIVWRIRK-NH_2_) [38], 1018 (VRLIVAVRIWRR-NH_2_) [39] and the D-enantiomer DJK-5 (VQWRAIRVRVIR-NH_2_) [25] were synthesized by CPC Scientific using solid-phase 9-flurenylmethoxy carbonyl (Fmoc) chemistry and purified to >95% purity using reverse-phase high-performance liquid chromatography (HPLC). The lyophilized peptides were resuspended in endotoxin-free water. The antibiotics gentamicin, ciprofloxacin, meropenem, erythromycin, clindamycin, vancomycin, azithromycin, and colistin were purchased from Sigma-Aldrich at a United States Pharmacopeia (USP) Reference Standard grade. Erythromycin and azithromycin were initially dissolved in 70% ethanol, while all other antibiotics were dissolved in endotoxin-free water (E-Toxate, Sigma-Aldrich). Antibiotics and peptides were further diluted into saline (Sigma-Aldrich) for *in vivo* application.

### Drug susceptibility

The MICs of drugs for all clinical isolates were determined by using the broth microdilution assay [40] in 96-well plates using Mueller-Hinton broth (MHB; Difco). All tests were performed in at least triplicate following the Clinical and Laboratory Standards Institute recommendations. Bacterial growth (37°C) was examined by visual inspection after 16 h to 48 h of incubation. The MIC was defined as the lowest concentration of a compound that completely prevented visible cell growth.

### Checkerboard titration assays

*P*. *aeruginosa* LESB58, LESB58.*relA*, LESB58.Δ*relA*/Δ*spoT*, and LESB58.Δ*relA*/Δ*spoT* (complement with plasmid pBBR5.*relA*) were adjusted to an OD_600_ of 0.001 and grown in a 96-well plate in MHB for 24 h at 37°C under static conditions. To chemically induce stringent conditions, 500 μM serine hydroxamate (SHX; Sigma-Aldrich) was added to the growth medium. The minimum fractional inhibitory concentration for each compound was visually determined in wells that showed 100% growth inhibition.

### Ethics statement

Animal experiments were performed in accordance with The Canadian Council on Animal Care (CCAC) guidelines and were approved by the University of British Columbia Animal Care Committee (certificate number A14-0363).

### Cutaneous mouse infection model

Mice used in this study were female outbred CD-1. All animals were purchased from Charles River Laboratories (Wilmington, MA), were 7 weeks of age, and weighed about 25 ± 3 g at the time of the experiments. 1 to 3% isoflurane was used to anesthetize the mice. Mice were euthanized with carbon dioxide.

The abscess infection model was performed as described earlier [4]. All microorganisms used in this infection model were grown to an OD_600_ of 1.0 in dYT broth. Prior to injection, bacterial cells were washed twice with sterile PBS and resuspended to the following (strain-dependent) concentrations to produce reproducible abscesses and bacterial counts: *P*. *aeruginosa* LESB58, 5 × 10^7^ CFU; *A*. *baumannii* Ab5075, 1 × 10^9^ CFU; *K*. *pneumoniae* KPLN49, 1 × 10^9^ CFU; *E*. *faecium* #1–1, 1 × 10^9^ CFU; *E*. *cloacae* 218R1, 2.5 × 10^8^ CFU; *E*. *coli* E38, 1 × 10^8^ CFU; and *S*. *aureus* LAC, 5 × 10^7^ CFU/ml (S5 Table). A 50 μl bacterial suspension was injected into the right side of the dorsum. All utilized peptides and antibiotics were tested for skin toxicity prior to efficacy testing. Treatment was applied directly into the subcutaneous space into the infected area (100 μl) at 1 h post infection. The progression of the disease/infection was monitored daily and abscesses (visible swollen, inflamed lumps) were measured on day three using a caliper. Skin abscesses were excised (including all accumulated pus), homogenized in sterile PBS using a Mini-Beadbeater-96 (Biospec products) for 5 min and bacterial counts determined by serial dilution. Experiments were performed at least 3 times independently with 2 to 4 animals per group.

### Tracking luminescence tagged bacteria during infection

To follow disease progress in real-time bioluminescently labelled strains were used (S5 Table). Bioluminescence images were acquired (auto exposure, medium binning) at different times after the initiation of infection by using the IVIS Lumina system (Perkin Elmer, Waltham MA) and analyzed using Living Image software.

### Outer membrane permeabilization assay

The induction of increases in the outer membrane permeability caused by antibiotics or peptides was evaluated using the fluorescence dye N-phenyl-1-naphthylamine (NPN; Sigma-Aldrich), based on the protocol of Loh et al [41]. Briefly, microtitre plates were prepared with 100 μl Hepes buffer (5 mM, pH 7.2) (control) or buffer supplemented with 0.5 mM NPN with or without peptides/antibiotics at a concentration equivalent to the MIC and 10-fold the MIC of individual bacterial strains. Bacterial strains were grown overnight on dYT agar plates, scraped from the plate, resuspended in buffer and adjusted to an OD_600_ of 1.0. One hundred μl of the cell suspension was then added to each well of the microtitre plate and the fluorescence immediately measured at an excitation wavelength of 350 nm and emission wavelength of 420 nm in a Synergy H1 microplate reader (BioTek). The values obtained from the cell suspension without test compounds were subtracted from the value for the suspension with test substrates to express the relative fluorescence units. All obtained data points were divided by 100 for presentation.

### Statistical analysis

Statistical evaluations were performed using GraphPad Prism 7.0 (GraphPad Software, La Jolla, CA, USA). *P*-values were calculated using one-way ANOVA, Kruskal-Wallis multiple-comparison test. Data was considered significant when *p*-values were below 0.05, 0.01 or 0.001 as indicated.

## Supporting information

S1 Table Antibiotics used in this study. (DOCX)

S2 Table Activity of tested antibiotics and peptides *in vivo* in the mouse abscess model. (DOCX)

S3 Table Influence of the stringent response on the combined efficacy of ciprofloxacin and peptides against *P*. *aeruginosa* LESB58 *in vitro*. The MIC values refer to the concentration required to give 100% inhibition of planktonic cell growth in MHB medium. Checkerboard titration experiments were performed to assess the synergistic interactions between DJK-5 or 1018 with ciprofloxacin.
(DOCX)

S4 Table Outer membrane permeabilization by peptides, cf. antibiotics, at 10-fold higher than their corresponding MICs. The uptake of the fluorophore NPN in the presence of different antibiotics and synthetic peptides was determined by assessing increased fluorescence at an excitation wavelength of 350 nm and an emission wavelength of 420 nm due to partition of the normally impermeable hydrophobic NPN into bacterial membranes. Relative fluorescence values of at least three biological replicates were determined by subtracting the fluorescence value without test substance.
(DOCX)

S5 Table Strains used in this study. (DOCX)

S6 Table Plasmids used in this study. (DOCX)

## References

[1] Santajit S, Indrawattana N. Mechanisms of antimicrobial resistance in ESKAPE pathogens. Biomed Res Int. 2016;2016:2475067 doi: 10.1155/2016/2475067 27274985 10.1155/2016/2475067 PMC4871955

[2] Khan HA, A A.; Mehboob R. Nosocomial infections and their control strategies. Asian Pac J Trop Dis. 2015;5(7):509–14.

[3] Udekwu KI, Parrish N, Ankomah P, Baquero F, Levin BR. Functional relationship between bacterial cell density and the efficacy of antibiotics. J Antimicrob Chemother. 2009;63(4):745–57. Epub 2009/02/17. doi: 10.1093/jac/dkn554 19218572 10.1093/jac/dkn554 PMC2654042

[4] Pletzer D, Mansour SC, Wuerth K, Rahanjam N, Hancock REW. New mouse model for chronic infections by Gram-negative bacteria enabling the study of anti-infective efficacy and host-microbe interactions. MBio. 2017;8(1). doi: 10.1128/mBio.00140-17 28246361 10.1128/mBio.00140-17 PMC5347345

[5] Giordano P, Weber K, Gesin G, Kubert J. Skin and skin structure infections: treatment with newer generation fluoroquinolones. Ther Clin Risk Manag. 2007;3(2):309–17. 18360639 10.2147/tcrm.2007.3.2.309 PMC1936312

[6] Ki V, Rotstein C. Bacterial skin and soft tissue infections in adults: A review of their epidemiology, pathogenesis, diagnosis, treatment and site of care. Can J Infect Dis Med Microbiol. 2008;19(2):173–84. 19352449 10.1155/2008/846453 PMC2605859

[7] Ramakrishnan K, Salinas RC, Agudelo Higuita NI. Skin and soft tissue infections. Am Fam Physician. 2015;92(6):474–83. 26371732

[8] Lee SY, Kuti JL, Nicolau DP. Antimicrobial management of complicated skin and skin structure infections in the era of emerging resistance. Surg Infect (Larchmt). 2005;6(3):283–95. doi: 10.1089/sur.2005.6.283 16201938 10.1089/sur.2005.6.283

[9] Raghavan M, Linden PK. Newer treatment options for skin and soft tissue infections. Drugs. 2004;64(15):1621–42. 15257625 10.2165/00003495-200464150-00002

[10] Rennie RP, Jones RN, Mutnick AH, Group SPS. Occurrence and antimicrobial susceptibility patterns of pathogens isolated from skin and soft tissue infections: report from the SENTRY Antimicrobial Surveillance Program (United States and Canada, 2000). Diagn Microbiol Infect Dis. 2003;45(4):287–93. 12730001 10.1016/s0732-8893(02)00543-6

[11] Ali A, Botha J, Tiruvoipati R. Fatal skin and soft tissue infection of multidrug resistant *Acinetobacter baumannii*: A case report. Int J Surg Case Rep. 2014;5(8):532–6. doi: 10.1016/j.ijscr.2014.04.019 25016080 10.1016/j.ijscr.2014.04.019 PMC4147652

[12] Konig C, Simmen HP, Blaser J. Bacterial concentrations in pus and infected peritoneal fluid—implications for bactericidal activity of antibiotics. J Antimicrob Chemother. 1998;42(2):227–32. 9738841 10.1093/jac/42.2.227

[13] Shukla C, Patel V, Juluru R, Stagni G. Quantification and prediction of skin pharmacokinetics of amoxicillin and cefuroxime. Biopharm Drug Dispos. 2009;30(6):281–93. doi: 10.1002/bdd.658 19591230 10.1002/bdd.658

[14] FDA M. Meropenem [package insert], MERREM I.V., https://www.accessdata.fda.gov/drugsatfda_docs/label/2008/050706s022lbl.pdf. 2017.

[15] Mansour SC, Pena OM, Hancock REW. Host defense peptides: front-line immunomodulators. Trends Immunol. 2014;35(9):443–50. doi: 10.1016/j.it.2014.07.004 25113635 10.1016/j.it.2014.07.004

[16] Haney EF, Hancock REW. Peptide design for antimicrobial and immunomodulatory applications. Biopolymers. 2013;100(6):572–83. doi: 10.1002/bip.22250 23553602 10.1002/bip.22250 PMC3932157

[17] de la Fuente-Nunez C, Reffuveille F, Haney EF, Straus SK, Hancock REW. Broad-spectrum anti-biofilm peptide that targets a cellular stress response. PLoS Pathog. 2014;10(5):e1004152 doi: 10.1371/journal.ppat.1004152 24852171 10.1371/journal.ppat.1004152 PMC4031209

[18] Pletzer D, Wolfmeier H, Bains M, Hancock REW. Synthetic peptides to target stringent response-controlled virulence in a *Pseudomonas aeruginosa* murine cutaneous infection model. Front Microbiol. 2017;8:1867 doi: 10.3389/fmicb.2017.01867 29021784 10.3389/fmicb.2017.01867 PMC5623667

[19] May JG, Shah P, Sachdeva L, Micale M, Kruper GJ, Sheyn A, et al Potential role of biofilms in deep cervical abscess. Int J Pediatr Otorhinolaryngol. 2014;78(1):10–3. Epub 2013/11/28. doi: 10.1016/j.ijporl.2013.09.009 24275082 10.1016/j.ijporl.2013.09.009

[20] Mansour SC, Pletzer D, de la Fuente-Nunez C, Kim P, Cheung GYC, Joo HS, et al Bacterial abscess formation is controlled by the stringent stress response and can be targeted therapeutically. EBioMedicine. 2016;12:219–26. doi: 10.1016/j.ebiom.2016.09.015 27658736 10.1016/j.ebiom.2016.09.015 PMC5078632

[21] Tangden T. Combination antibiotic therapy for multidrug-resistant Gram-negative bacteria. Ups J Med Sci. 2014;119(2):149–53. doi: 10.3109/03009734.2014.899279 24666223 10.3109/03009734.2014.899279 PMC4034552

[22] Tamma PD, Cosgrove SE, Maragakis LL. Combination therapy for treatment of infections with gram-negative bacteria. Clin Microbiol Rev. 2012;25(3):450–70. doi: 10.1128/CMR.05041-11 22763634 10.1128/CMR.05041-11 PMC3416487

[23] Fantin B, Carbon C. In vivo antibiotic synergism: contribution of animal models. Antimicrob Agents Chemother. 1992;36(5):907–12. 1510412 10.1128/aac.36.5.907 PMC188745

[24] Scholar EM, Pratt WB. The antimicrobial drugs: Determinants of bacterial response to antimicrobial agents. 2nd ed New York: Oxford University Press; 2000 xii, 607 p. p.

[25] de la Fuente-Nunez C, Reffuveille F, Mansour SC, Reckseidler-Zenteno SL, Hernandez D, Brackman G, et al D-enantiomeric peptides that eradicate wild-type and multidrug-resistant biofilms and protect against lethal *Pseudomonas aeruginosa* infections. Chem Biol. 2015;22(2):196–205. doi: 10.1016/j.chembiol.2015.01.002 25699603 10.1016/j.chembiol.2015.01.002 PMC4362967

[26] Poole K. Bacterial stress responses as determinants of antimicrobial resistance. J Antimicrob Chemother. 2012;67(9):2069–89. doi: 10.1093/jac/dks196 22618862 10.1093/jac/dks196

[27] Andersson DI, Hughes D. Antibiotic resistance and its cost: is it possible to reverse resistance? Nat Rev Microbiol. 2010;8(4):260–71. doi: 10.1038/nrmicro2319 20208551 10.1038/nrmicro2319

[28] Nguyen D, Joshi-Datar A, Lepine F, Bauerle E, Olakanmi O, Beer K, et al Active starvation responses mediate antibiotic tolerance in biofilms and nutrient-limited bacteria. Science. 2011;334(6058):982–6. doi: 10.1126/science.1211037 22096200 10.1126/science.1211037 PMC4046891

[29] Amato SM, Orman MA, Brynildsen MP. Metabolic control of persister formation in *Escherichia coli*. Mol Cell. 2013;50(4):475–87. doi: 10.1016/j.molcel.2013.04.002 23665232 10.1016/j.molcel.2013.04.002

[30] Mohamed YF, Abou-Shleib HM, Khalil AM, El-Guink NM, El-Nakeeb MA. Membrane permeabilization of colistin toward pan-drug resistant Gram-negative isolates. Braz J Microbiol. 2016;47(2):381–8. doi: 10.1016/j.bjm.2016.01.007 26991296 10.1016/j.bjm.2016.01.007 PMC4874589

[31] Behroozian S, Svensson SL, Davies J. Kisameet clay exhibits potent antibacterial activity against the ESKAPE pathogens. MBio. 2016;7(1):e01842–15. doi: 10.1128/mBio.01842-15 26814180 10.1128/mBio.01842-15 PMC4742703

[32] Jacobs AC, Thompson MG, Black CC, Kessler JL, Clark LP, McQueary CN, et al AB5075, a highly virulent isolate of *Acinetobacter baumannii*, as a model strain for the evaluation of pathogenesis and antimicrobial treatments. MBio. 2014;5(3):e01076–14. doi: 10.1128/mBio.01076-14 24865555 10.1128/mBio.01076-14 PMC4045072

[33] Cheng K, Smyth RL, Govan JR, Doherty C, Winstanley C, Denning N, et al Spread of beta-lactam-resistant *Pseudomonas aeruginosa* in a cystic fibrosis clinic. Lancet. 1996;348(9028):639–42. doi: 10.1016/S0140-6736(96)05169-0 8782753 10.1016/S0140-6736(96)05169-0

[34] Marchou B, Bellido F, Charnas R, Lucain C, Pechere JC. Contribution of beta-lactamase hydrolysis and outer membrane permeability to ceftriaxone resistance in *Enterobacter cloacae*. Antimicrob Agents Chemother. 1987;31(10):1589–95. 3501699 10.1128/aac.31.10.1589 PMC174996

[35] Centers for Disease C, Prevention. Outbreaks of community-associated methicillin-resistant *Staphylococcus aureus* skin infections-Los Angeles County, California, 2002–2003. MMWR Morb Mortal Wkly Rep. 2003;52(5):88. 12588006

[36] Kovach ME, Elzer PH, Hill DS, Robertson GT, Farris MA, Roop RM 2nd, et al Four new derivatives of the broad-host-range cloning vector pBBR1MCS, carrying different antibiotic-resistance cassettes. Gene. 1995;166(1):175–6. 8529885 10.1016/0378-1119(95)00584-1

[37] Cherkasov A, Hilpert K, Jenssen H, Fjell CD, Waldbrook M, Mullaly SC, et al Use of artificial intelligence in the design of small peptide antibiotics effective against a broad spectrum of highly antibiotic-resistant superbugs. ACS Chem Biol. 2009;4(1):65–74. doi: 10.1021/cb800240j 19055425 10.1021/cb800240j

[38] Nijnik A, Madera L, Ma S, Waldbrook M, Elliott MR, Easton DM, et al Synthetic cationic peptide IDR-1002 provides protection against bacterial infections through chemokine induction and enhanced leukocyte recruitment. J Immunol. 2010;184(5):2539–50. doi: 10.4049/jimmunol.0901813 20107187 10.4049/jimmunol.0901813

[39] Achtman AH, Pilat S, Law CW, Lynn DJ, Janot L, Mayer ML, et al Effective adjunctive therapy by an innate defense regulatory peptide in a preclinical model of severe malaria. Sci Transl Med. 2012;4(135):135ra64 doi: 10.1126/scitranslmed.3003515 22623740 10.1126/scitranslmed.3003515

[40] Wiegand I, Hilpert K, Hancock REW. Agar and broth dilution methods to determine the minimal inhibitory concentration (MIC) of antimicrobial substances. Nat Protoc. 2008;3(2):163–75. doi: 10.1038/nprot.2007.521 18274517 10.1038/nprot.2007.521

[41] Loh B, Grant C, Hancock REW. Use of the fluorescent probe 1-N-phenylnaphthylamine to study the interactions of aminoglycoside antibiotics with the outer membrane of *Pseudomonas aeruginosa*. Antimicrob Agents Chemother. 1984;26(4):546–51. 6440475 10.1128/aac.26.4.546 PMC179961

