# Supplementary material for: Synergy between conventional antibiotics and anti-biofilm peptides in a murine, sub-cutaneous abscess model caused by recalcitrant ESKAPE pathogens
Source: PLoS Pathog. 2018 Jun 21;14(6):e1007084. doi: 10.1371/journal.ppat.1007084 (PMC6013096; doi:10.1371/journal.ppat.1007084)
Supplement: S1 Table — (DOCX) [file ppat.1007084.s001.docx]

# S1 Table: Antibiotics used in this study

**EUCAST clinical breakpoint information for all tested antibiotics**^a^

| **Strain** | **EUCAST Clinical Breakpoints^b^** | | | | | | | | | | | |
| --- | --- | --- | --- | --- | --- | --- | --- | --- | --- | --- | --- | --- |
|  | **AZA**^c^ | **AZM** | **CAM** | **CAZ** | **CIP** | **COL** | **ERY** | **GEN** | **MERO** | **RIF** | **TOB** | **VAN** |
| *A. baumannii* Ab5075 | NA | NA | NA | NA | R | S | NA | R | R | NA | R | NA |
| *E. coli* E38 | NA | NA | NA | S | S | S | NA | R | S | NA | S | NA |
| *E. cloacae* 218R1 | NA | NA | NA | R | S | S | NA | S | S | NA | S | NA |
| *E. faecium* #1-1 | NA | NA | NA | NA | R | NA | NA | NA | NA | NA | NA | R |
| *K. pneumoniae* KPN649 | NA | NA | NA | R | R | S | NA | R | S | NA | R | NA |
| *P. aeruginosa* LESB58 | NA | NA | NA | R | R | R | NA | R | S | NA | R | NA |
| *S. aureus* LAC | NA | R | NA | NA | R | NA | R | R | NA | S | S | S |

**EUCAST breakpoints for administered antibiotics (MIC breakpoint (mg/L))**^b^

| **Strain** | **ERY^c^** | **GEN** | **MERO** | **VAN** | **CIP** | **CLI** |
| --- | --- | --- | --- | --- | --- | --- |
| *A. baumannii* Ab5075 | NA | S<=4; R>4 | S<=2; R>8 | NA | S<=1; R>1 | NA |
| *E. coli* E38 | NA | S<=2; R>4 | S<=2; R>8 | NA | S<=0.5; R>1 | NA |
| *E. cloacae* 218R1 | NA | S<=2; R>4 | S<=2; R>8 | NA | S<=0.25; R>0.5 | NA |
| *E. faecium* #1-1 | NA | NA | NA | S<=4; R>4 | S<=4; R>4 | NA |
| *K. pneumoniae* KPN649 | NA | S<=2; R>4 | S<=2; R>8 | NA | S<=0.5; R>1 | NA |
| *P. aeruginosa* LESB58 | NA | S<=4; R>4 | S<=2; R>8 | NA | S<=0.5; R>0.5 | NA |
| *S. aureus* LAC | S<=1; R>2 | S<=1; R>1 | NA | S<=2; R>2 | S<=1; R>1 | S<=0.25; R>0.5 |

^a^ http://www.eucast.org/clinical_breakpoints (website version 8.0 accessed Apr. 13, 2018)

^b^ S, sensitive; R, resistant; NA, not applicable

^c^ AZA, Aztreonam; AZM, Azithromycin; CAM, Chloramphenicol; CAZ, Ceftazidime; CIP, Ciprofloxacin; COL, Colistin; ERY, Erythromycin; GEN, Gentamicin; MERO, Meropenem; RIF, Rifampicin; TOB, Tobramycin; VAN, Vancomycin

**Pharmacological information**

| **Antibiotic** | **Class** | **Mechanism of action** | **Dose IV** | **Mean peak serum concentration**^d^ | **Reference** |
| --- | --- | --- | --- | --- | --- |
| Ciprofloxacin | Fluoroquinolone | Inhibits DNA gyrase, and topoisomerase type IV | 400 mg | 4.6 μg/ml | [[40](#_ENREF_40)] |
| Erythromycin | Macrolide | Binds 50S subunit of rRNA; protein synthesis inhibitor | 500 mg | 10 μg/ml | [[41](#_ENREF_41)] |
| Meropenem | Carbapenem | Cell wall synthesis inhibitor | 500 mg | 23 μg/ml^e^ | [[13](#_ENREF_13)] |
| Gentamicin | Aminoglycoside | Binds 30S subunit of ribosomes; protein synthesis inhibitor | 1 mg/kg^c^ | >4 μg/ml | [[42](#_ENREF_42)] |
| Clindamycin | Lincosamide | Binds 50S subunit of rRNA; protein synthesis inhibitor | 900 mg | 14.1 μg/ml^f^ | [[43](#_ENREF_43)] |
| Vancomycin | Glycopeptide | Gram-positive cell wall synthesis inhibitor | 1000 mg | 63 μg/ml | [[44](#_ENREF_44)] |

^d^ 1 h IV infusion

^e^ 30 min IV infusion

^f^ applied intramuscular (mean peak concentration up to four times the single IM dose)

**References**

40. FDA C. Ciprofloxacin [package insert], CIPRO® IV, <https://www.accessdata.fda.gov/drugsatfda_docs/label/2013/019857s062lbl.pdf>. 2017.

41. FDA E. Erythromycin [package insert], Erythrocin™ Lactobionate - IV, <https://www.accessdata.fda.gov/drugsatfda_docs/label/2013/050609s034lbl.pdf>. 2017.

42. FDA G. Gentamicin [package insert], <https://www.accessdata.fda.gov/drugsatfda_docs/label/2014/062366s033lbl.pdf>. 2017.

43. FDA C. Clindamycin [package insert], Cleocin Phosphate®, <https://www.accessdata.fda.gov/drugsatfda_docs/label/2008/050441s055,050639s016lbl.pdf>. 2017.

44. FDA V. Vancomycin [package insert], Vancomycin Injection, USP, <https://www.accessdata.fda.gov/drugsatfda_docs/label/2015/050671s022lbl.pdf>. 2017.
