# Supplementary material for: Synergy between conventional antibiotics and anti-biofilm peptides in a murine, sub-cutaneous abscess model caused by recalcitrant ESKAPE pathogens
Source: PLoS Pathog. 2018 Jun 21;14(6):e1007084. doi: 10.1371/journal.ppat.1007084 (PMC6013096; doi:10.1371/journal.ppat.1007084)
Supplement: S2 Table — (DOCX) [file ppat.1007084.s002.docx]

# S2 Table: Activity of tested antibiotics and peptides *in vivo* in the mouse abscess model. Antibiotic abbreviations as per S1 Table.

| **Strain / Treatment** | **Area of dermonecrosis (mm^2^)** | | | **CFU per abscess** | |
| --- | --- | --- | --- | --- | --- |
|  | Mean | ± SE | Fold-change^a^ | Geometric mean | Fold-change^a^ |
| ***P. aeruginosa* LESB58** |  |  |  |  |  |
| Saline | 76.2 | 4.6 | - | 4.2 × 10^8^ | - |
| DJK-5 (3 mg/kg) | 47.7 | 5.6 | -1.6 | 9.0 × 10^7^ | -4.6 |
| 1018 (10 mg/kg) | 35.9 | 4.9 | -2.1 | 1.7 × 10^8^ | -2.4 |
| CIP (0.4 mg/kg) | 26.8 | 4.2 | -2.8 | 2.7 × 10^7^ | -15.4 |
| DJK-5 (3 mg/kg), CIP (0.4 mg/kg) | 20.1 | 4.8 | -3.8 | 1.7 × 10^6^ | -244.6 |
| 1018 (10 mg/kg), CIP (0.4 mg/kg) | 8.1 | 2.6 | -9.4 | 2.2 × 10^6^ | -185.9 |
| Combined single treatment DJK-5, CIP | 37.9 | 4.3 | -1.9^b^ | 5.1 × 10^7^ | -29.9^b^ |
| Combined single treatment 1018, CIP | 31.4 | 3.3 | -3.9^b^ | 6.7 × 10^7^ | -30.4^b^ |
| ***E. faecium #1-1*** |  |  |  |  |  |
| Saline | 34.8 | 3.6 | - | 5.7 × 10^6^ | - |
| DJK-5 (3 mg/kg) | 22.2 | 1.5 | -1.6 | 2.6 × 10^5^ | -22.2 |
| 1002 (10 mg/kg) | 16.0 | 1.9 | -2.2 | 3.5 × 10^6^ | -1.6 |
| Gm (16 mg/kg) | 20.9 | 2.4 | -1.7 | 1.5 × 10^6^ | -3.9 |
| DJK-5 (3 mg/kg), GEN (16 mg/kg) | 3.3 | 0.4 | -10.6 | 2.2 × 10^4^ | -264.7 |
| 1002 (10 mg/kg), GEN (16 mg/kg) | 8.3 | 1.3 | -4.2 | 3.1 × 10^5^ | -18.2 |
| Combined single treatment DJK-5, GEN | 21.5 | 1.4 | -6.6^b^ | 6.2 × 10^5^ | -28.6^b^ |
| Combined single treatment 1002, GEN | 18.6 | 1.7 | -2.3^b^ | 2.2 × 10^6^ | -7.1^b^ |
| ***K. pneumoniae* KPLN649** |  |  |  |  |  |
| Saline | 41.1 | 1.8 | - | 7.7 × 10^8^ | - |
| DJK-5 (3 mg/kg) | 39.6 | 2.3 | -1.0 | 1.9 × 10^8^ | -4.0 |
| 1018 (10 mg/kg) | 31.1 | 3.0 | -1.3 | 9.4 × 10^8^ | -0.8 |
| MERO (10 mg/kg) | 39.6 | 4.4 | -1.0 | 2.8 × 10^8^ | -2.7 |
| CIP (30 mg/kg) | 33.1 | 4.3 | -1.2 | 3.2 × 10^8^ | -2.4 |
| DJK-5 (3 mg/kg), MERO (10 mg/kg) | 29.5 | 5.6 | -1.4 | 8.5 × 10^6^ | -90.9 |
| 1018 (10 mg/kg), CIP (30 mg/kg) | 29.1 | 3.8 | -1.4 | 1.1 × 10^8^ | -6.9 |
| Combined single treatment DJK-5, MERO | 37.5 | 2.6 | -1.3^b^ | 2.3 × 10^8^ | -27.6^b^ |
| Combined single treatment 1018, CIP | 32.1 | 2.5 | -1.1^b^ | 7.1 × 10^8^ | -6.4^b^ |
| ***A. baumannii* Ab5075** |  |  |  |  |  |
| Saline | 32.4 | 1.7 | - | 4.7 × 10^7^ | - |
| DJK-5 (3 mg/kg) | 19.9 | 2.1 | -1.6 | 4.7 × 10^6^ | -9.9 |
| MERO (6 mg/kg) | 26.6 | 5.2 | -1.2 | 4.1 × 10^5^ | -111.1 |
| ERY (6 mg/kg) | 19.0 | 2.5 | -1.7 | 2.9 × 10^5^ | -161.5 |
| DJK-5 (3 mg/kg), ERY (6 mg/kg) | 21.9 | 3.0 | -1.5 | 3.5 × 10^4^ | -1325.1 |
| DJK-5 (3 mg/kg), MERO (6 mg/kg) | 32.6 | 6.4 | -1.0 | 2.3 × 10^4^ | -2006.5 |
| Combined single treatment DJK-5, ERY | 19.4 | 1.6 | -0.9^b^ | 1.2 × 10^6^ | -33.1^b^ |
| Combined single treatment DJK-5, MERO | 23.1 | 2.7 | -0.7^b^ | 1.5 × 10^6^ | -64.9^b^ |
| ***E. coli* E38** |  |  |  |  |  |
| Saline | 64.2 | 9.2 | - | 9.1 × 10^8^ | - |
| DJK-5 (3 mg/kg) | 74.5 | 5.4 | -0.9 | 4.1 × 10^8^ | -2.2 |
| 1018 (10 mg/kg) | 48.1 | 10.4 | -1.3 | 6.9 × 10^8^ | -1.3 |
| CIP (4 mg/kg) | 77.6 | 13.0 | -0.8 | 1.6 × 10^8^ | -5.8 |
| DJK-5 (3 mg/kg), CIP (4 mg/kg) | 20.2 | 6.4 | -3.2 | 8.9 × 10^7^ | -10.3 |
| 1018 (10 mg/kg), CIP (4 mg/kg) | 24.8 | 5.5 | -2.6 | 1.1 × 10^8^ | -8.0 |
| Combined single treatment DJK-5, CIP | 81.9 | 8.7 | -4.1^b^ | 2.5 × 10^8^ | -2.9^b^ |
| Combined single treatment 1018, CIP | 68.3 | 10.4 | -2.8^b^ | 3.3 × 10^8^ | -2.9^b^ |
| ***E. cloacae* 218R1** |  |  |  |  |  |
| Saline | 93.8 | 10.9 | - | 4.6 × 10^8^ | - |
| 1018 (10 mg/kg) | 95.7 | 16.9 | -1.0 | 4.0 × 10^8^ | -1.1 |
| HHC-10 (10 mg/kg) | 68.8 | 20.0 | -1.4 | 1.6 × 10^8^ | -2.9 |
| CIP (0.006 mg/kg) | 39.1 | 9.2 | -2.4 | 1.7 × 10^8^ | -2.7 |
| 1018 (10 mg/kg), CIP (0.006 mg/kg) | 23.3 | 4.5 | -4.0 | 8.6 × 10^7^ | -5.4 |
| HHC-10 (10 mg/kg), CIP (0.006 mg/kg) | 14.0 | 5.0 | -6.7 | 1.3 × 10^7^ | -36.1 |
| Combined single treatment 1018, CIP | 67.4 | 11.6 | -2.9^b^ | 1.8 × 10^8^ | -2.2^b^ |
| Combined single treatment HHC-10, CIP | 53.9 | 11.3 | -3.9^b^ | 1.7 × 10^8^ | -13.1^b^ |
| ***S. aureus* LAC** |  |  |  |  |  |
| Saline | 146.2 | 16.66 | - | 7.2 × 10^7^ | - |
| DJK-5 (0.25 mg/kg) | 152.9 | 24.31 | -1.0 | 1.1 × 10^8^ | -0.7 |
| CLI (0.01 mg/kg) | 64.21 | 9.172 | -2.3 | 7.7 × 10^7^ | -0.9 |
| VAN (0.15 mg/kg) | 75.11 | 17.81 | -1.9 | 5.8 × 10^7^ | -1.2 |
| DJK-5 (0.25 mg/kg) / CLI (0.01 mg/kg) | 45.51 | 14.33 | -3.2 | 5.3 × 10^7^ | -1.4 |
| DJK-5 (0.25 mg/kg) / VAN (0.15 mg/kg) | 18.52 | 5.475 | -7.9 | 6.6 × 10^6^ | -10.9 |
| Combined single treatment DJK-5, CLI | 114 | 18.06 | -2.5^b^ | 7.8 × 10^7^ | -1.5^b^ |
| Combined single treatment DJK-5, VAN | 108.6 | 17.53 | -5.9^b^ | 9.0 × 10^7^ | -13.7^b^ |

^a^ compared to Saline control

^b^ compared to combined treatment
