# Supplementary material for: Synergy between conventional antibiotics and anti-biofilm peptides in a murine, sub-cutaneous abscess model caused by recalcitrant ESKAPE pathogens
Source: PLoS Pathog. 2018 Jun 21;14(6):e1007084. doi: 10.1371/journal.ppat.1007084 (PMC6013096; doi:10.1371/journal.ppat.1007084)
Supplement: S3 Table — The MIC values refer to the concentration required to give 100% inhibition of planktonic cell growth in MHB medium. Checkerboard titration experiments were performed to assess the synergistic interactions between DJK-5 or 1018 with ciprofloxacin. (DOCX) [file ppat.1007084.s003.docx]

# S3 Table: Influence of the stringent response on the combined efficacy of ciprofloxacin and peptides against *P. aeruginosa* LESB58 *in vitro*. The MIC values refer to the concentration required to give 100% inhibition of planktonic cell growth in MHB medium. Checkerboard titration experiments were performed to assess the synergistic interactions between DJK-5 or 1018 with ciprofloxacin.

| **LESB58 Strains** | **Peptide** | **Ciprofloxacin MIC (μg/ml)** | **Peptide MIC (μg/ml)** | **Fold decrease in antibiotic concentration in combination** | **Fold decrease in peptide concentration in combination** |
| --- | --- | --- | --- | --- | --- |
| WT | 1018 | 3.13 | 50 | 0X | 2X |
| WT + 500 μM SHX | 1018 | 3.13 | 50 | 2X | 4X |
| WT with cloned, overexpressed *relA*^+^ | 1018 | 3.13 | 50 | 2X | 4X |
| Δ*relA*Δ*spoT* double mutant | 1018 | 1.56 | 12.5 | 0X | 0X |
| Δ*relA*Δ*spoT* double mutant + 500 μM SHX | 1018 | 1.56 | 12.5 | 0X | 0X |
| Δ*relA*Δ*spoT* complemented with cloned, overexpressed *relA*^+^ | 1018 | 6.25 | 25 | 2X | 4X |
| WT | DJK-5 | 3.13 | 50 | 0X | 2X |
| WT + 500 μM SHX | DJK-5 | 3.13 | 50 | 2X | 4X |
| WT with cloned, overexpressed *relA*^+^ | DJK-5 | 3.13 | 50 | 2X | 4X |
| Δ*relA*Δ*spoT* double mutant | DJK-5 | 1.56 | 25 | 0X | 0X |
| Δ*relA*Δ*spoT* double mutant + 500 μM SHX | DJK-5 | 1.56 | 25 | 0X | 0X |
| Δ*relA*Δ*spoT* complemented with cloned, overexpressed *relA*^+^ | DJK-5 | 6.25 | 50 | 2X | 4X |
