# Supplementary material for: Synergy between conventional antibiotics and anti-biofilm peptides in a murine, sub-cutaneous abscess model caused by recalcitrant ESKAPE pathogens
Source: PLoS Pathog. 2018 Jun 21;14(6):e1007084. doi: 10.1371/journal.ppat.1007084 (PMC6013096; doi:10.1371/journal.ppat.1007084)
Supplement: S4 Table — The uptake of the fluorophore NPN in the presence of different antibiotics and synthetic peptides was determined by assessing increased fluorescence at an excitation wavelength of 350 nm and an emission wavelength of 420 nm due to partition of the normally impermeable hydrophobic NPN into bacterial membranes. Relative fluorescence values of at least three biological replicates were determined by subtracting the fluorescence value without test substance. (DOCX) [file ppat.1007084.s004.docx]

**S4 Table: Outer membrane permeabilization by peptides cf. antibiotics at 10-fold higher than their corresponding MICs.** The uptake of the fluorophore NPN in the presence of different antibiotics and synthetic peptides was determined by assessing increased fluorescence at an excitation wavelength of 350 nm and an emission wavelength of 420 nm due to partition of the normally impermeable hydrophobic NPN into bacterial membranes. Relative fluorescence values of at least three biological replicates were determined by subtracting the fluorescence value without test substance.

| **Strain** | Relative NPN Fluorescence (Mean ± Standard Error) | | | | | |
| --- | --- | --- | --- | --- | --- | --- |
|  | **Antibiotic^a^** | **1018** | **HHC-10** | **DJK-5** | **1002** | **Colistin** |
| *A. baumannii* Ab5075 | 0 ± 0 | 110 ± 4.0^d^ | 73 ± 1.5 | 11 ± 3.0^d^ | 45 ± 9.1 | 101 ± 16^d^ |
| *E. coli* E38 | 18 ± 17 | 68 ± 9^d^ | 48 ± 3.0 | 58 ± 18 | 22 ± 10 | 91 ± 12^d^ |
| *E. cloacae* 218R1 | 0 ± 0 | 101 ± 21^d^ | 77 ± 4.6^d^ | 51 ± 16 | 2.1 ± 1.2 | 111 ± 7.2^d^ |
| *K. pneumoniae* KPN649 | 13 ± 11 | 92 ± 17^d^ | 87 ± 23^d^ | 36 ± 15 | 4.6 ± 4.6 | 105 ± 15^d^ |
| *P. aeruginosa* LESB58 | 0.6 ± 0.6 | 333 ± 140^d^ | 350 ± 158 | 353 ± 164^d^ | 49 ± 28 | 369 ± 150^d^ |
| *E. faecium* #1-1 | 0 ± 0^b^ | 2.2 ± 2.2 | 0 ± 0 | 0 ± 0 | 0 ± 0 | 0 ± 0 |
| *S. aureus* LAC | 1.0 ± 1.0^c^ | 4.3 ± 3.3 | 0 ± 0 | 0 ± 0 | 0 ± 0 | 12 ± 12 |

^a^ Meropenem was used unless indicated otherwise

^b^ Gentamicin

^c^ Vancomycin

^d^ Significant difference to the antibiotic (p < 0.05; One-Way ANOVA, Kruskal-Wallis test).
