# Supplementary material for: Synergy between conventional antibiotics and anti-biofilm peptides in a murine, sub-cutaneous abscess model caused by recalcitrant ESKAPE pathogens
Source: PLoS Pathog. 2018 Jun 21;14(6):e1007084. doi: 10.1371/journal.ppat.1007084 (PMC6013096; doi:10.1371/journal.ppat.1007084)
Supplement: S5 Table — (DOCX) [file ppat.1007084.s005.docx]

# S5 Table: Strains used in this study

| **Strains** | **Relevant characteristics or genotype^a^** | **Injection inoculum^b^** | **Reference or source** |
| --- | --- | --- | --- |
| ***Pseudomonas aeruginosa*** |  |  |  |
| LESB58 | Liverpool Epidemic Strain isolate | 1 × 10^9^ | [[31](#_ENREF_31)] |
| LESB58.lux | Ap^r^, Gm^r^; carrying pUCP::*luxCDABE* | 1 × 10^9^ | [[11](#_ENREF_11)] |
| LESB58/relA^+^ | Gm^r^; carrying pBBR5.relA |  | This study |
| LESB58 Δ*relA*Δ*spoT* | Δ*relA*/Δ*spoT* double deletion mutant |  | [17] |
| LESB58 Δ*relA*Δ*spoT* (*relA^+^*) | Δ*relA*/Δ*spoT* deletion mutant chromosomally complemented with the *relA* gene including its promoter region |  | [17] |
| ***Acinetobacter baumannii*** |  |  |  |
| Ab5075 | Highly virulent clinical wound isolate | 2 × 10^10^ | [[30](#_ENREF_30)] |
| Ab5075.lux | Tc^r^; carrying pBBR3::*luxCDABE* | 2 × 10^10^ | This study |
| ***Enterococcus faecium*** |  |  |  |
| #1-1 | Stool isolate from a human patient prior to bacteremia | 2 × 10^10^ | BEI Resources |
| #1-1.lux | Spc^r^; carrying pSL101_P16S_ | 2 × 10^10^ | This study |
| ***Klebsiella pneumoniae*** |  |  |  |
| KPLN49 | Wild type strain | 2 × 10^10^ | [[29](#_ENREF_29)] |
| KPLN49.lux | Cm^r^; carrying pBBR1::*luxCDABE* | 2 × 10^10^ | This study |
| ***Enterobacter cloacae*** |  |  |  |
| 218R1 | Class C chromosomal β-lactamase overproducing strain | 5 × 10^9^ | [[32](#_ENREF_32)] |
| 218R1.lux | Gm^r^; carrying pBBR5::*luxCDABE* | 5 × 10^9^ | This study |
| ***Escherichia coli*** |  |  |  |
| E38 (serotype O78:H-) | Human peritoneum isolate | 2 × 10^9^ | BEI Resources |
| E38.lux | Tc^r^; carrying pBBR3.lux | 2 × 10^9^ | This study |
| ***S. aureus*** |  |  |  |
| LAC USA300 | Community-associated methicillin-resistant (CA-MRSA) strain USA300 | 5 × 10^7^ | [[33](#_ENREF_33)] |
| LAC.lux | Cm^r^; Ap^r^; Ery^r^; carrying pRP1195::*luxCDABE* | 5 × 10^7^ | [[45](#_ENREF_45)] |

^a^ Antibiotic resistance: Ap^r^, ampicillin; Cm^r^, chloramphenicol; Gm^r^, gentamicin; Tc^r^, tetracycline; Km^r^, kanamycin; Spc^r^, spectinomycin; Ery^r^, erythromycin.

^b^ CFU/ml

**References**

45. Plaut RD, Mocca CP, Prabhakara R, Merkel TJ, Stibitz S. Stably luminescent *Staphylococcus aureus* clinical strains for use in bioluminescent imaging. PLoS One. 2013;8(3):e59232.
