# Supplementary material for: Synergy between conventional antibiotics and anti-biofilm peptides in a murine, sub-cutaneous abscess model caused by recalcitrant ESKAPE pathogens
Source: PLoS Pathog. 2018 Jun 21;14(6):e1007084. doi: 10.1371/journal.ppat.1007084 (PMC6013096; doi:10.1371/journal.ppat.1007084)
Supplement: S6 Table — (DOCX) [file ppat.1007084.s006.docx]

# S6 Table: Plasmids used in this study

| **Plasmid** | **Relevant characteristics^a^** | **Reference or source** |
| --- | --- | --- |
| pUCP.lux | Ap^r^, Gm^r^; pUCP23 containing *luxCDABE* operon | [[46](#_ENREF_46)] |
| pCR-BluntII-TOPO | Km^r^, Zc^r^; Zero Blunt cloning vector | Invitrogen |
| pTOPO.lux | Km^r^, Zc^r^; contains 5.8 kb *luxCDABE* operon fused to a t0 transcriptional terminator sequence | This study |
| pBBR1MCS-1 | Cm^r^; broad-host-range cloning vector | [[34](#_ENREF_34)] |
| pBBR1.lux | Cm^r^; contains *luxCDABE*-t0 under *lac* promoter control | This study |
| pBBR1MCS-3 | Tc^r^; broad-host-range cloning vector | [[34](#_ENREF_34)] |
| pBBR3.lux | Tc^r^; contains *luxCDABE*-t0 under *lac* promoter control | This study |
| pBBR1MCS-5 | Gm^r^; broad-host-range cloning vector | [[34](#_ENREF_34)] |
| pBBR5.lux | Gm^r^; contains *luxCDABE*-t0 under *lac* promoter control | This study |
| pBBR5.relA | Gm^r^; contains *relA* under *lac* promoter control | This study |
| pSL101_P16S_ | Spc^r^; contains synthetic 16S promoter fused to lux genes | [[47](#_ENREF_47)] |

^a^ Antibiotic resistance: Ap^r^, ampicillin, Zc^r^, zeocin; Cm^r^, chloramphenicol; Gm^r^, gentamicin; Tc^r^, tetracycline; Km^r^, kanamycin; Spc^r^, spectinomycin.

**References**

46. McPhee JB, Lewenza S, Hancock REW. Cationic antimicrobial peptides activate a two-component regulatory system, PmrA-PmrB, that regulates resistance to polymyxin B and cationic antimicrobial peptides in *Pseudomonas aeruginosa*. Mol Microbiol. 2003;50(1):205-17.

47. La Rosa SL, Diep DB, Nes IF, Brede DA. Construction and application of a *luxABCDE* reporter system for real-time monitoring of *Enterococcus faecalis* gene expression and growth. Appl Environ Microbiol. 2012;78(19):7003-11.
